# Supplementary material for: Complex‐centric proteome profiling by SEC‐SWATH‐MS
Source: Mol Syst Biol. 2019 Jan 14;15(1):e8438. doi: 10.15252/msb.20188438 (PMC6346213; doi:10.15252/msb.20188438)
Supplement: Supplementary file 8 — Dataset EV7 [file MSB-15-e8438-s008.zip › feature_plots_string/P30405.pdf]

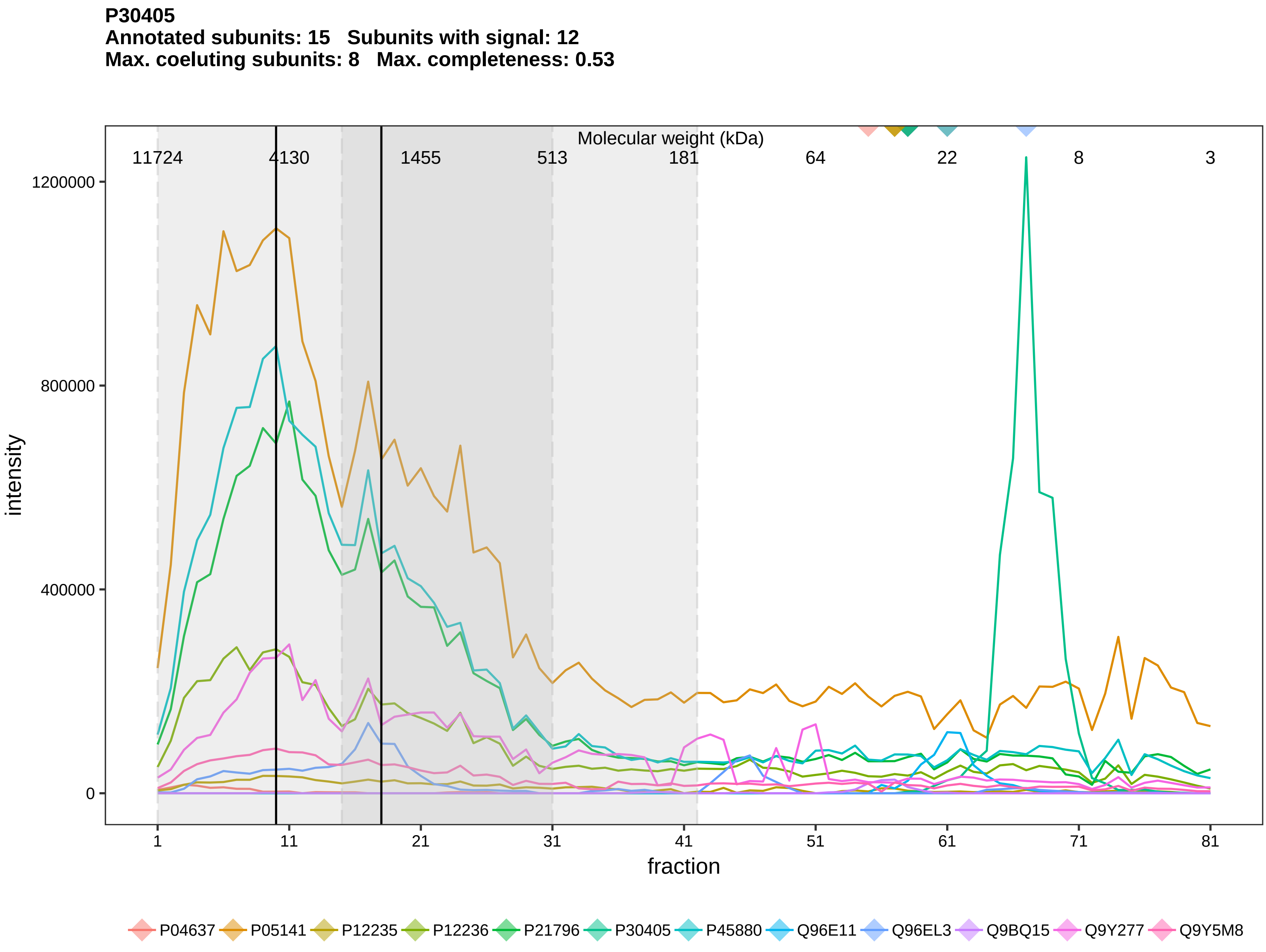

P30405
Annotated subunits: 15 Subunits with signal: 12
Max. coeluting subunits: 8 Max. completeness: 0.53
Molecular weight (kDa)
11724
4130
1455
513
181
64
22
8
3
1200000
800000
400000
0
intensity
1
11
21
31
41
51
61
71
81
fraction
P04637
P05141
P12235
P12236
P21796
P30405
P45880
Q96E11
Q96EL3
Q9BQ15
Q9Y277
Q9Y5M8
